# Induction of selective cytotoxicity and apoptosis in human T4-lymphoblastoid cell line (CEMss) by boesenbergin a isolated from *boesenbergia rotunda* rhizomes involves mitochondrial pathway, activation of caspase 3 and G2/M phase cell cycle arrest

**DOI:** 10.1186/1472-6882-13-41

**Published:** 2013-02-22

**Authors:** Kuan-Beng Ng, Ahmad Bustamam, Mohd Aspollah Sukari, Siddig Ibrahim Abdelwahab, Syam Mohan, Michael James Christopher Buckle, Behnam Kamalidehghan, Nabilah Muhammad Nadzri, Theebaa Anasamy, A Hamid A Hadi, Heshu Sulaiman Rahman

**Affiliations:** 1 UPM-MAKNA Cancer Research Laboratory, Institute of Bioscience, Universiti Putra Malaysia, Serdang, Selangor, Malaysia; 2 Department of Chemistry, Faculty of Science, Universiti Putra Malaysia, Serdang, SelangorMalaysia; 3 Medical Research Centre, Jazan University, P.O. Box 114, Jazan, Kingdom of Saudi Arabia; 4 Department of Pharmacy, Faculty of Medicine, University of Malaya, Kuala Lumpur, 50603, Malaysia; 5 Faculty of Science, University of Malaya, Kuala Lumpur, 50603, Malaysia; 6 Faculty of Veterinary Medicine, Universiti Putra Malaysia, Serdang, Selangor, Malaysia

**Keywords:** Boesenbergia rotunda, Boesenbergin A, CEMss, Anticancer, Cytotoxicity

## Abstract

**Background:**

*Boesenbergia rotunda* (Roxb.) Schlecht (family zingiberaceae) is a rhizomatous herb that is distributed from north-eastern India to south-east Asia, especially in Indonesia, Thailand and Malaysia. Previous research has shown that the crude extract of this plant has cytotoxic properties. The current study examines the cytotoxic properties of boesenbergin A isolated from *Boesenbergia rotunda*.

**Methods:**

MTT assay was used to check the cytotoxicity of boesenbergin A. The morphological assessment of apoptosis was monitored using normal and fluorescence microscopy. The early and late phase of apoptosis was investigated using annexin V and DNA laddering assays, respectively. The mitochondrial membrane potential (MMP) was assessed by fluorescence microscopy. Human apoptosis proteome profiler assays were performed to investigate the mechanism of cell death. In addition, the protein levels of Bax, Bcl2 and HSP 70 were also analyzed using western blot. Assays of caspase =-3/7, -8 and =-9 were carried out in order to test for induction during treatment. Lastly, cell cycle progression was analyzed using flow cytometry.

**Results:**

Boesenbergin A was found to have the highest toxicity towards CEMss cancer cells (IC_50_ = 8 μg/ml). The morphology of CEMss cells after treatment showed evidence of apoptosis that included blebbing and chromatin condensation. The annexin V assay revealed that early apoptosis is induced after treatment. The DNA laddering assay confirmed that DNA fragmentation had occurred during late apoptosis. The cell cycle analysis indicated that boesenbergin A was able to induce G2/M phase arrest in CEMss cells. The activity of caspases -3/7, -8 and -9 was increased after treatment which indicates both intrinsic and extrinsic pathways are induced during apoptosis. The involvement of mitochondria was established by increased mitochondrial membrane potential and up and down regulation of Bcl2 and Bax proteins as well as HSP70.

**Conclusion:**

In conclusion, the results demonstrated that boesenbergin A induced apoptosis of CEMss cells through Bcl2/Bax signaling pathways with the involvement of caspases and G2/M phase cell cycle arrest. The current findings warrant further research on boesenbergin A as a novel chemotherapeutic agent for leukemia intervention including studies in animal models.

## Background

Leukemia is a cancer of blood-forming organs, such as bone marrow, which is characterized by the uncontrolled proliferation of abnormal blood cells [1]. In a national childhood cancer survey carried out in Malaysia, it was found that leukemia is the commonest childhood tumor. The crude incidence rate of pediatric malignancies in Malaysia was 77.4 per million children aged less than 15 years with leukemia as the fourth leading cause of cancer related death in 1998 [2].

There are about 20,000 species of tropical plants, of which about 1,300 are said to be medicinal and potential sources for screening of anticancer agents [3]. Some of the plant extracts from these medicinal plants are reported to have potential to be developed as drugs [4]. *Boesenbergia rotunda* (L.) (Fingerroot), formerly known as *Boesenbergia* or *Kaempferia pandurata* (Roxb). Schltr. (Zingiberaceae), is distributed in south-east Asian countries, such as Indonesia, Malaysia and Thailand. The rhizomes of this plant have been used for the treatment of peptic ulcer, as well as colic, oral diseases, urinary disorders, dysentery and inflammation [5]. Several studies have suggested this plant to be neuroprotective and to show anti-inflammatory, anti-mutagenic, anticancer, chemopreventive, anti-dermatophytic, *anti-Helicobacter pylori* and anti-dengue-2 virus NS3 protease activity [6]. This plant possesses both anti-oxidant, as well as anticancer properties which can help to cure cancer.

**Figure 1:**
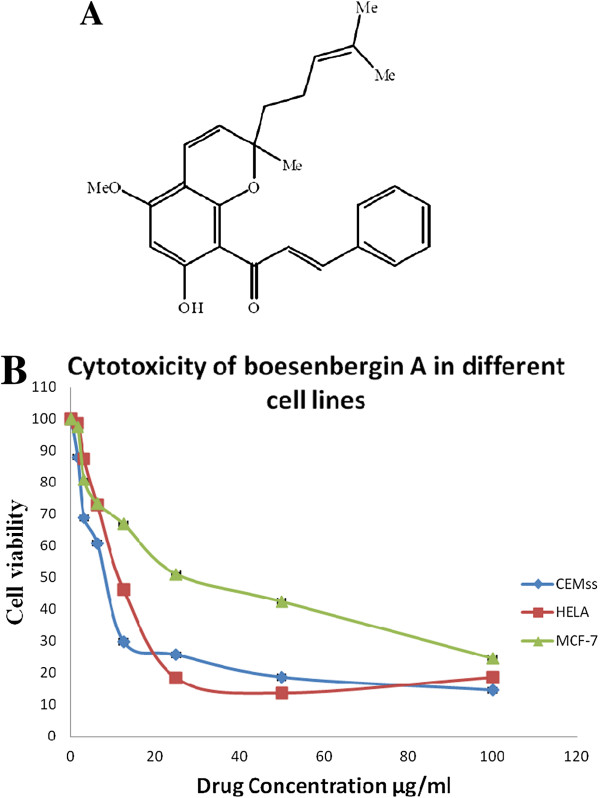
(A) Structure of Boesenbergin A and (B) Cytotoxicity of BA in different cell lines.

Among the compounds that have been isolated from *Boesenbergia rotunda* are flavonoids, pinocembrin and alpinetin, and chalcones, panduratin A, cardamonin and boesenbergin A [7,8]. There are varieties of important biological compounds central core are formed by aromatic ketones from chalcones. The central core contains two aromatic rings with an unsaturated chain and it shows antibacterial, antifungal, chemopreventive, antiviral, antiprotozoal, insecticidal, anticancer, and anti-inflammatory properties [9-11]. One of the chalcones that are already being studied by quite a number of scientists is panduratin A. Of these panduratin A has been shown to be capable of inducing apoptosis and cell cycle arrest in human prostate cancer cells PC3 and DU145 [12] and of inhibiting the growth of A549 cells through induction of apoptosis and inhibition of NF-kB translocation [13]. On the other hand, only preliminary studies of the cytotoxic activity of boesenbergin A against HL-60 cells have been reported [14]. Following on from recent findings in our research group that boesenbergin A also has cytotoxic activity against A549, PC3, HepG2, HT-29, and WRL-68 cells [15], the present study boesenbergin A (Figure 1) explores its anticancer potential in human acute lymphoblastic leukemia cells (CEMss) *in vitro*.

## Methods

### Plant materials

The rhizomes of B. rotunda were purchased from Puchong Market, Selangor Darul Ehsan, Malaysia. The material was identified by a botanist at the Faculty of Science, University Putra Malaysia, where a voucher specimen was deposited (BR-R11-01). The isolation and identification of boesenbergin A has been previously reported by us in detail [14].

### Cell culture conditions

Estrogen receptor positive cells (MCF-7) and cervical cancer cells (Hela) were obtained from ATCC (USA). Human T4-lymphoblastoid cell line CEMss were obtained from NIH AIDS Research and Reference Reagent Program (Division of AIDS, NIAID, NIH: USA). The cell lines were grown at 37°C in a humidified CO_2_ incubator with 5% CO_2_ in RPMI-1640 (Sigma, MO, USA) supplemented with 10% fetal bovine serum (Invitrogen Corp., Auckland, N.Z.).

### Cytotoxicity assay

Adherent cells (1× 10^6^ cells/ml) were grown in 96-well plates overnight, whereas CEMss cells (1× 10^6^ cells/ml) were plated directly into 96-well plates on the drug treatment day. Boesenbergin A was dissolved in dimethylsulfoxide (DMSO) and media. The final concentration of DMSO was 0.1% (v/v). Different concentrations of the sample were prepared with serial dilution. Dimethylsulfoxide (0.1%) was used as a control. The toxicity profiles of the compound were assessed using the 3-[4,5-dimethylthiazol-2-yl]-2,5-diphenyltetrazolium bromide (MTT) microculture tetrazolium viability assay as described previously [16]. Thereafter, various concentrations of compound (with a maximum of 100 μg/ml) were plated out in triplicate. Each plate included untreated cell controls and a blank cell-free control. After 72 h of incubation, MTT (5 mg/ml) was added to each well and the plates incubated for a further 4 h before removal of the media. DMSO was then added into each well to solubilize the formazan crystals. The absorbance was read at wavelength of 595 nm using a microtitre plate reader (Labsystems iEMS Reader MF). The percentage cellular viability was calculated with the appropriate controls taken into account. The concentration which inhibited 50% of cellular growth (IC_50_ value) was determined. All experiments were carried out in triplicate (Figure 1B).

The inhibitory rate of cell proliferation was calculated by the following formula: Growth inhibition = (OD control – OD treated) / OD control X 100. The cytotoxicity of sample on cancer cells was expressed as IC_50_ values (the drug concentration reducing the absorbance of treated cells by 50% with respect to untreated cells). The determined IC_50_ value was used for many of the subsequent experiments.

### Cytotoxicity of boesenbergin A on proliferated primary human blood lymphocytes

The ability of boesenbergin A to act selectively on cancer cells especially leukemia was evaluated by comparing the cytotoxicity of this compound towards primary human blood lymphocytes. Briefly, blood was collected into a cell preparation tube containing sodium citrate (BD Vacutainer®, New Jersey, USA). After collection, tube was stood upright for 20 min at room temperature to allow it to equilibrate and was then centrifuged at 1200xg for 20 min. Mononuclear cells and platelets underneath the plasma layer were collected using a pipette and transferred into a 15 ml centrifuge tube. The cells were washed twice with PBS and cultured in complete Quantum PBL media with phytohemagglutinin (PAA, Pasching, Austria) containing 10% FBS supplemented with 100 U/ml penicillin and 100 μg/ml streptomycin at 37°C in 5% CO_2_ atmosphere. Primary human blood lymphocytes (1 × 10^6^ cells/ml) were treated at various concentrations of boesenbergin A in triplicate and cell viability was measured using MTT assay after 48 h of incubation. The cell lines used as normal cells were human peripheral blood lymphocytes obtained from normal healthy donors after informed consent was given. This project was approved by the medical research ethics committee (founded in 2002) of the medical Faculty of UPM at a meeting on April 12, 2012 (UPM 2564/004/12).

### Microscopic observation of cellular morphology using phase contrast inverted microscope

This analysis examined whether apoptosis may be implicated in mediating cell death amongst CEMss cells elicited by boesenbergin A. CEMss cells at a concentration of 1 × 10^6^ cells/ml were cultured in RPMI 1640 (PAA, Cölbe, Germany) medium containing 10% FBS was seeded into a 25 ml culture flask (TPP, Trasadingen, Switzerland) and treated with boesenbergin A (8 μg/ml) at different time periods (24, 48 and 72 h). The morphological appearance of treated cells was compared with the untreated control by using a normal inverted microscope post-treatment [17]. CEMss cells were treated with the compound for 24, 48 and 72 h. Untreated cells served as the negative control.

### Quantification of apoptosis using propidium iodide and acridine orange double staining

Boesenbergin A-induced cell death in CEMss cells was quantified using propidium iodide (PI) and acridine orange (AO) double staining according to standard procedures and examined under a fluorescence microscope (Lieca attached with Q-Floro Software). Briefly, treatment was carried out in a 25 ml culture flask (Nunc). CEMss cells were plated at a concentration of 1 × 10^6^ cells/ml and treated with boesenbergin A (8 μg/ml). The cells were incubated in 5% CO_2_ atmosphere at 37°C for 24, 48 and 72 h and then spun down at 200 g for 10 min. The supernatant was discarded and the cells were washed twice using PBS after centrifuging at 200 g for 10 min to remove the remaining media. Ten microliters of fluorescent dyes containing AO (10 μg/ml) and PI (10 μg/ml) were added into the cellular pellet at equal volumes of each. The freshly stained cell suspension was dropped into a glass slide and covered by cover slip. Slides were observed under a UV-fluorescence microscope within 30 min before the fluorescence colour started to fade. The percentages of viable, early apoptotic, late apoptotic and secondary necrotic cells were determined in more than 200 cells. Acridine orange (AO) and propidium iodide (PI) are intercalating nucleic acid specific fluorochromes which emit green and orange fluorescences respectively, when they are bound to DNA. Of the two, only AO can cross the plasma membrane of viable and early apoptotic cells. The criteria for identification are as follows: (a) viable cells appear to have a green nucleus with an intact structure; (b) early apoptotic cells exhibit a bright-green nucleus showing condensation of chromatin in the nucleus; (c) late apoptotic cells show dense orange areas of chromatin condensation; (d) secondary necrotic cells appear to have an orange intact nucleus [18]. This assay provides a useful quantitative evaluation and was carried out in triplicate.

### Flow cytometric analysis of DNA cell cycle

CEMss cells at concentration of 1 × 10^6^ cells/ml were cultured in RPMI 1640 (PAA) medium containing 10% FBS seeded in to 25 ml culture flask (TPP) and treated with boesenbergin A (8 μg/ml) at different time periods (24, 48 and 72 h). After incubation, the cells were spun down by centrifugation at 200 g for 10 min. The supernatant was discarded and the pellet was washed with PBS twice to remove any remaining media. To restore the integrity, fixation of the cell population for flow cytometery analysis was performed. Briefly, cell pellets were fixed by mixing 500 μl of 70% cold ethanol and 250 μl of cell suspension and kept at −20°C overnight. The cells were then spun down at 200 g for 10 min and the ethanol was decanted. After washing twice with PBS, the cells were resuspended in PBS. Twenty microliters of RNase A (10 μg/ml) and 2 μl of PI (2.5 μg/ml) were added and the fixed cells were kept in the dark on ice for 30 min. Propidium iodide has the ability to bind to RNA molecules and hence RNase enzyme was added in order to allow PI to bind directly to DNA. The DNA content of cells was then analyzed using a flow cytometer (BD FACSCanto™ II). The fluorescence intensity of the subG_1_ cell fraction represents the apoptotic cell population.

### Annexin V assay

CEMss cells (1 × 10^6^ cells/ml) were exposed to boesenbergin A (8 μg/ml) for 24, 48 and 72 h and the annexin V assay performed using an annexin V:FITC assay kit (ABD Serotec, UK). Briefly the treated cells were centrifuged for 10 min at 200 g to remove the media. After that, PBS was added to wash the cells and the same process was repeated twice. Then 5 μl annexin V:FITC was added to 195 μl of the cell suspension binding buffer, which was prepared by diluting the binding buffer 1:4 in distilled water (50 ml binding buffer +150 ml distilled water). The suspension was then mixed and incubated for 10 min in the dark at room temperature. The cells were then washed and resuspended in 190 μl prediluted binding buffer. Then 10 μl of the PI solution was added to the cell suspension and the sample was analyzed using a flow cytometer (BD FACSCanto™ II).

### DNA laddering

The Apoptotic DNA Ladder Detection kit (CHEMICON International Inc., CA, USA) was used for DNA extraction from cells. Briefly, CEMss cells treated with boesenbergin A (16 μg/ml) were collected at 24 and 48 h post treatment. The cells were washed with PBS and the cells were spun down by centrifugation at 500 × g for 5 min. After removal of the supernatant, the cells were lysed by the addition of 40 μl of TE lysis buffer and gentle pipetting, followed by the addition of 5 μl of Enzyme A (RNase A) into the crude lysate and incubated at 37°C for 10 min. Then 5 μl of Enzyme B (Proteinase K) was added and the lysate was further incubated at 50°C for 30 min. Then 5 μl of ammonium acetate solution and 50 μl of isopropanol were added and mixed well and the suspension was kept at −20°C for 10 min. The sample was then centrifuged 16000 × g for 10 min at to precipitate the DNA. After washing the DNA pellet with 70% ice cold ethanol, the air dried pellet was dissolved in 30 μl of DNA suspension buffer. For detecting the DNA ladder, the extracted DNA samples were run on 1% agarose gel in tris–acetic acid–EDTA buffer. After electrophoresis, the gel was stained with ethidium bromide (Gibco BRL, Paisley, Scotland), visualized with a UV light transilluminator (UVP, Upland, CA, USA) and photographed.

### Caspase-3/7, -8 and -9 activity assay

Assays of caspase-3/7, -8 and =-9 was performed using the Caspase-Glo Assay kit (Promega, WI, USA). CEMss cells were plated and treated with boesenbergin A (8 μg/ml) and incubated for 24, 48 and 72 h in 96 well white plates. After allowing the cells to equilibriate at room temperature, 50 μl of Caspase-Glo® reagent was added to each well containing 50 μl of blank, negative control cells and treated cells in culture medium. The contents of the plate were gently mixed using a plate shaker at 100 g for 30 sec. It was then incubated at room temperature for 30 min in the dark. Readings were taken every 10 min for 3 h using a luminescence microplate reader (Infinite M200PRO, Tecan, Männedorf, Switzerland).

### Detection of mitochondrial membrane potential (Δψm)

Rhodamine 123 (Rh123) is a fluorescent cationic dye that binds to polarized mitochondrial membrane and accumulates as aggregates in the mitochondria of normal cells. Rh123 was prepared in ethanol as a 5 mg/ml stock solution. CEMss cells were treated with 8 μg/ml boesenbergin A for 24, 48 and 72 h. At the end of the reaction time, the cells were harvested and washed twice in cold PBS, then resuspended in Rh123 (2 μg/ml) for 30 min in the dark. The Rh123 staining intensity was captured using a fluorescence microscope. Intensity of Rh 123 is directly related to mitochondrial membrane potential. The percentage of rhodamine negative cells gives the percentage collapse of Mitochondria Membrane Permeability.

### Human apoptosis proteome profiler array

To investigate the pathways by which boesenbergin A induces apoptosis, we performed a determination of apoptosis-related proteins using the Proteome Profiler Array (RayBio® Human Apoptosis Antibody Array Kit, RayBiotech, GA, USA), according to the manufacturer’s instructions. Briefly, the cells where treated with boesenbergin A. Three hundred micrograms of protein from each sample were incubated with the human apoptosis array overnight. The apoptosis array data was quantified by scanning the membrane on a Biospectrum AC ChemiHR 40 (UVP, Upland, CA, USA) and analysis of the array image file was performed using image analysis software according to the manufacturer’s instructions.

### Western blot analysis

CEMss cells were seeded in 12-well plates and treated with boesenbergin A (8 μg/ml) at 3, 6, 12 and 24 h. The total protein of the cells was extracted with cell lysis buffer (50 mM Tris–HCl pH 8.0, 120 mM NaCl, 0.5% NP-40, 1 mM PMSF). Forty micrograms of protein extract was separated by 10% SDS-PAGE, transferred to a polyvinylidenedifluoride (PVDF) membrane (Bio-Rad), blocked with 5% nonfat milk in TBS-Tween buffer 7 (0.12 M Tris-base, 1.5 M NaCl, 0.1% Tween20) for 1 h at room temperature, incubated with the appropriate antibody overnight at 4°C and then incubated with horseradish peroxidase conjugated secondary antibody for 30 min at room temperature. The bound antibody was detected with peroxidase-conjugated anti-rabbit antibody (1:10000) or anti-mouse antibody (1:10000) followed by chemiluminescence (ECL System) and exposed by autoradiography. The following primary antibodies β-actin (1:10000), Bcl2 (1:1000), Bax (1:1000), HSP70 (1:1000), were purchased from Santa Cruz Biotechnology, Inc, (California, USA).

### Statistical analysis

Data is reported as the mean ± SD of three replicates. The independent *t*-test and ANOVA were used for comparisons with P < 0.05 considered to be significant. All statistical analyses were performed using the SPSS software (Release 18, SPSS Inc, Chicago, IL, USA).

## Results

### Cell growth cytotoxic assay

Several human cancer cell lines were used to screen the cytotoxicity of the boesenbergin A. The IC_50_ value on the viability of CEMss cells was determined to be 8.11 ± 0.44 μg/ml (20.07 μM). In addition, boesenbergin A also showed toxicity towards MCF-7 and Hela cells at IC_50_ values of 25.43 ± 0.36 μg/ml (62.95 μM) and 12.21 ± 0.28 μg/ml (30.22 μM), respectively (Table 1). The positive control used, 5-fluouracil produced an inhibitory effect on CEMss cells with an IC_50_ value of 1.43 ± 0.06 μg/ml. The results revealed that boesenbergin A demonstrated the highest toxicity towards CEMss cells. Hence CEMss cells were selected and used to further analyse the cytotoxic potential of boesenbergin A*.*

**Table 1:** Effect of boesenbergin A on the viability of MCF-7, Hela and CEMss cells for 72 h

**Cell type**	**IC **_**50 **_**± SD (μg /ml)**
MCF-7	25.43 ± 0.36
Hela	12.21 ± 0.28
CEMss	8.11 ± 0.44

Each value represents means ± SD.

### Cytotoxicity of boesenbergin A on proliferated primary human blood lymphocytes

From experiments carried out for 48 h, it was found that boesenbergin A has no toxic effect against primary human blood lymphocytes (Table 2).

**Table 2:** Effect of boesenbergin A on proliferated primary human blood lymphocyte for 48 h

**Boesenbergin A concentration (μg /ml)**	**Percentage viable cells (%)**
100	99
50	98
25	102
12.5	100
6.25	106
3.125	98
1.56	93
Control	100

### Microscopic observation of cellular morphology using phase contrast inverted microscope

**Figure 2:**
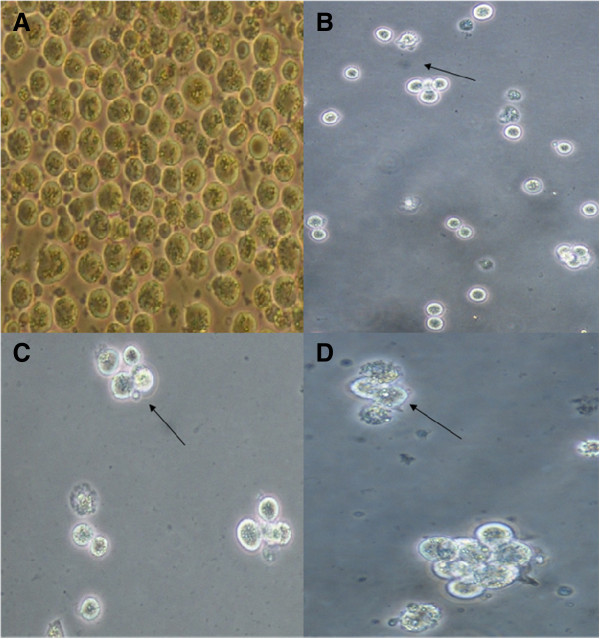
**Microscopic observation of cellular morphology using phase contrast inverted microscope of CEMss cells.** Cells were treated at IC_50_ Boesenbergin A in time-dependent manner. (**A**) Untreated cells showed normal structure without prominent apoptosis induction and necrosis. (**B**) Early apoptosis features were seen after 24 h representing (arrows) (**C**) Blebbing were noticed in 48 h treatment (arrows). (**D**) Increasing blebbing with chromatin condensation was seen during late apoptosis after 72 h incubation of CEMss with Boesenbergin A (arrows).

The study revealed that treatment of CEMss cells with boesenbergin A triggered morphological changes, which indicates that apoptosis occurred in a time-dependent manner. Figure 2 shows several morphological changes of treated and untreated CEMss cells at 24, 48 and 72 h post-treatment under 400 X magnification. Treated CEMss cells showed obvious changes indicating induction of apoptosis as compared to untreated cells. These features included blebbing of the cell membrane, prominent growth inhibition such as chromatin condensation and cell shrinkage. On the contrary, untreated cells remained confluent throughout the incubation period.

### Quantification of apoptosis using propidium iodide and acridine orange double staining

**Figure 3:**
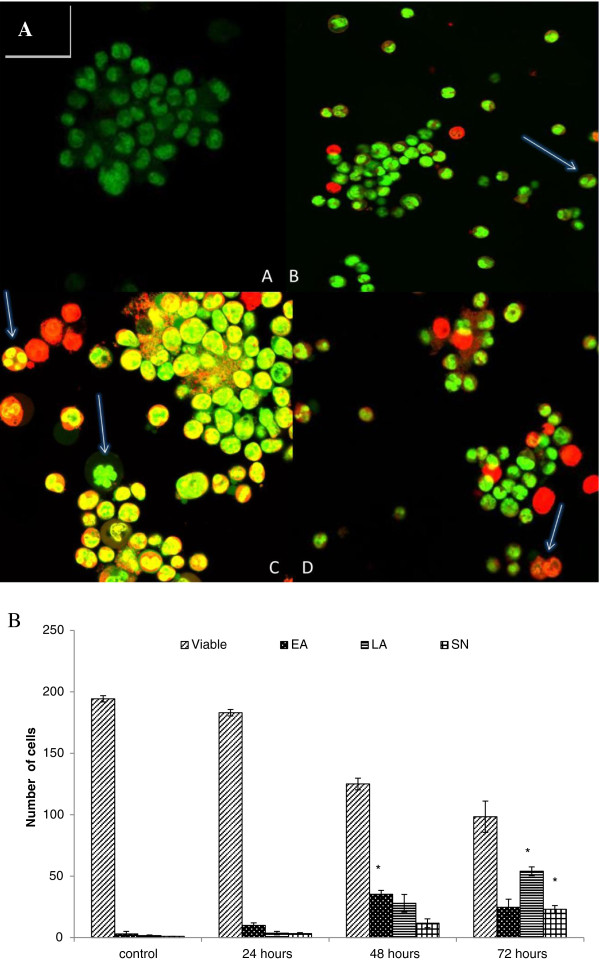
**Confocal micrograph of acridine orange and propidium iodide double-stained CEMss cells.** Cells were treated at IC_50_ of Boesenbergin A at time-dependent manner. (**A**) Untreated cells showed normal structure without prominent apoptosis and necrosis. (**B**) Early apoptosis features were seen after 24 h representing intercalated acridine orange (bright green) amongst the fragmented DNA (arrows). (**C**) Blebbing and nuclear margination were noticeable after 48 h treatment (arrows). (**D**) Late apoptosis was seen after 72 h post-treatment whereby a positive staining of orange color represents hallmark of late apoptosis (arrows). Figure 3B Histogram representing qualitative analysis of confocal micrograph consisting of acridine orange and propidium iodide double-stained treated and untreated CEMss cells. CEMss cell death via apoptosis increased significantly (p < 0.05) in a time-dependent manner. However, no significant (p > 0.05) difference was observed in the cell count of necrosis. (EA: early apoptosis; LA: late apoptosis; SN: secondary necrosis). ‘*’ indicates significant differences from the control (p < 0.05).

In order to quantify the degree of apoptosis, propidium iodide (PI) and acridine orange (AO) double-staining was used in this experiment. CEMss cells were scored under a confocal microscope after treatment, in order to quantify the number of cells that are categorized as viable, early apoptotic, late apoptotic and secondary necrotic. A total of 200 cells were used arbitrarily and differentially, together with an untreated negative control for scoring. The study revealed that boesenbergin A triggered morphological changes in treated CEMss cells that indicated possible induction of apoptosis upon treatment in a time dependent manner. The presence of intercalated AO within fragmented DNA indicates early apoptosis. At 24 h after treatment with boesenbergin A*,* blebbing and nuclear chromatin condensation were noticeable. Late apoptosis is indicated by the presence of reddish orange colour due to the binding of AO to denatured DNA as observed after 48 h treatment (Figure 3A). Differential scoring of treated CEMss cells (200 cells population) showed that there is a statistically significant (P < 0.05) difference in apoptosis positive cells. On the other hand, there was no statistically significant (P > 0.05) difference in necrotic counts at different treatment times (Figure 3B).

### Cell cycle analysis

**Figure 4:**
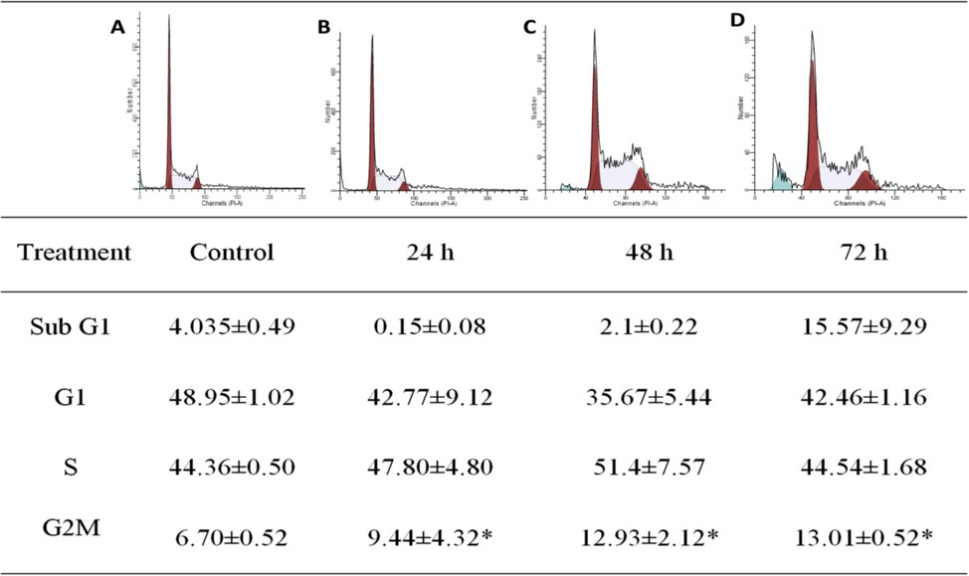
**Flow cytometric analysis of cell cycle distribution in CEMss cells treated with Boesenbergin A at 8 μg/ml for A) Untreated, B) 24, C) 48, and D) 72 h.** Data were shown as Mean ± SEM. *p<0.05 *vs.* 0 h (Control). Cells were cultured in RPMI 1640 (25 ml flask) media maintained at 37°C and 5% CO_2_.

Flow cytometric analysis of the cell cycle and DNA content were performed to determine the ability of boesenbergin A to induce cell cycle arrest and apoptosis. There were no significant changes of G1 and S in dose-dependent treatment on CEMss cells. However, the sub G1 phase, (apoptotic cells) showed a significant increase in a time dependent manner (Figure 4). These results suggest that boesenbergin A is capable of inducing significant G_2_/M phase arrest to CEMss cells.

### Annexin V assay

**Figure 5:**
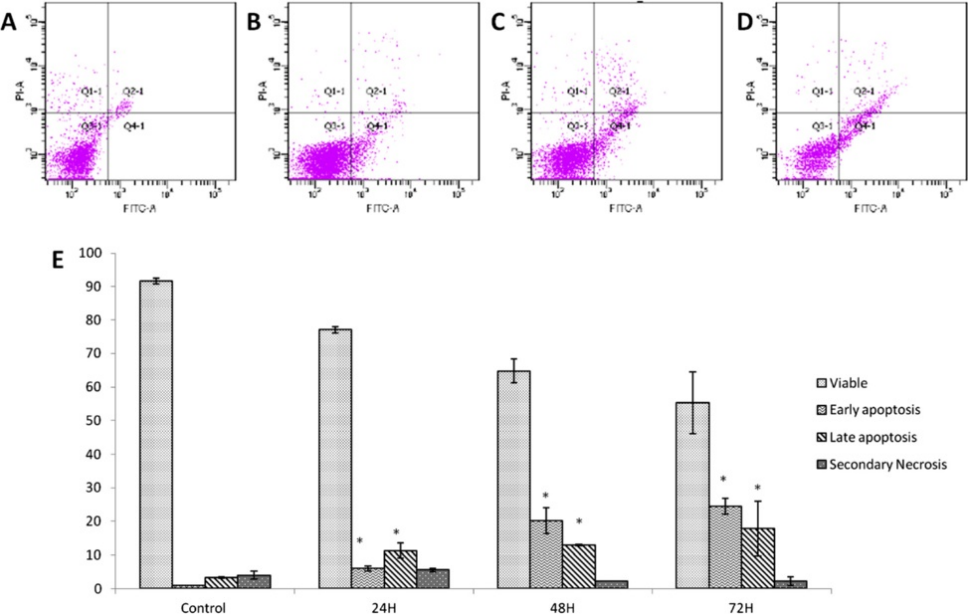
Graph of flow cytometric analysis of Annexin V in CEMss cells which were treated with Boesenbergin A 8 μg/ml for A) Untreated B) 24 h, C) 48 h and D) 72 h E) histogram.

The annexin V assay revealed the induction of apoptosis in CEMss cells at an early stage after treatment with 8 μg/ml of boesenbergin A. Negative control cells showed 91.7% viability, 1.0% in early apoptosis, 3.25% in late apoptosis and 4.0% in secondary necrosis, whereas after 24 h treatment with boesenbergin A, CEMss cells showed 77.05% viability, 6.05% in early apoptosis, 11.3% in late apoptosis and 5.55% in secondary necrosis. As the treatment time increased to 48 h and 72 h, the percentage of both early and late apoptotic cells continued to increase substantially (Figure 5). This result provided evidence that treatment of CEMss cells with boesenbergin A showed the presence of early apoptosis, late apoptosis and secondary necrosis in CEMss cells as the period of treatment was increased from 24 h to 72 h.

### DNA laddering

**Figure 6:**
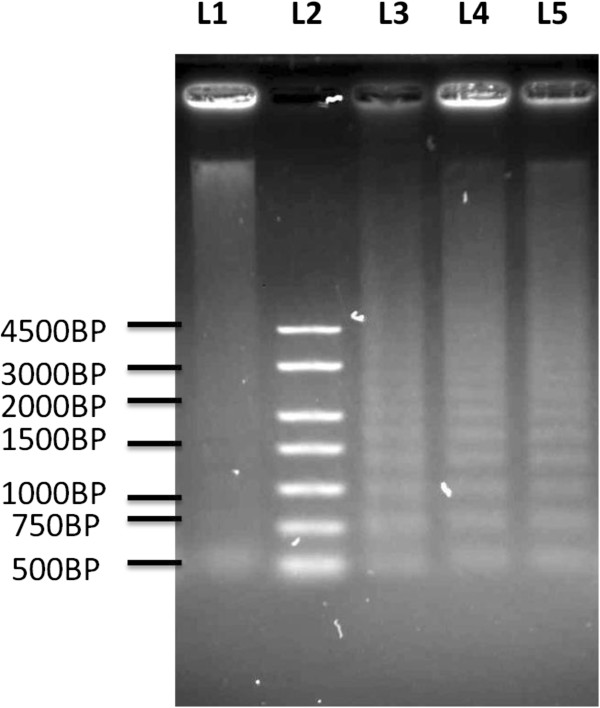
**Electrophoresis separation of fragmented DNA of untreated and treated CEMss cells for 24 h and 48 h with 16 μg/ml of Boesenbergin A.** Lane 1: Untreated cells. Lane 2: 250 base pair marker. Lane 3: Positive control which is HL-60 cells treated with actinomycin. Lane 4: CEMss cells treated with 16 μg/ml of Boesenbergin A for 24 h. Lane 5: CEMss cells treated with 16 μg/ml of Boesenbergin A for 48 h.

DNA fragmentation analysis, following treatment of CEMss cells with boesenbergin A (16 μg/ml) for 24 and 48 h*,* clearly showed evidence of DNA laddering in CEMss cells after treatment for 24 and 48 h. This appearance of DNA laddering is similar to the positive control of HL-60 cells treated with actinomycin (Figure 6). Untreated CEMss cells did not provide evidence of laddering. The appearance of DNA laddering after treatment of CEMss cells with boesenbergin A further confirms apoptosis, due to nuclear fragmentation.

### Caspase-3/7, -8 and =-9 analyses

**Figure 7:**
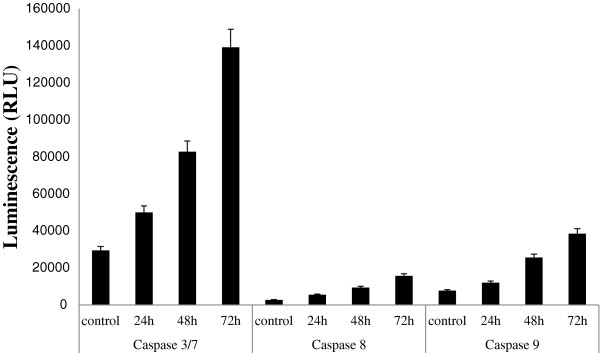
**The colourimetric assay of caspase -3/7, 8, and 9 in untreated and CEMss cells treated with Boesenbergin A 8 μg/ml for 24, 48 and 72 h.** Independent t-test showed a significance (p< 0.05) between control and treated cell activity of caspase-3/7, -8 and -9.

The assays of caspases =-3/7, -8 and =-9 showed that boesenbergin A significantly enhanced their activity in CEMss cells compared to untreated cells over a 24 to 72 h time period (Figure 7), thus implying that the compound induces apoptosis in CEMss cells through both intrinsic and also extrinsic pathways.

### Mitochondrial membrane potential analysis

**Figure 8:**
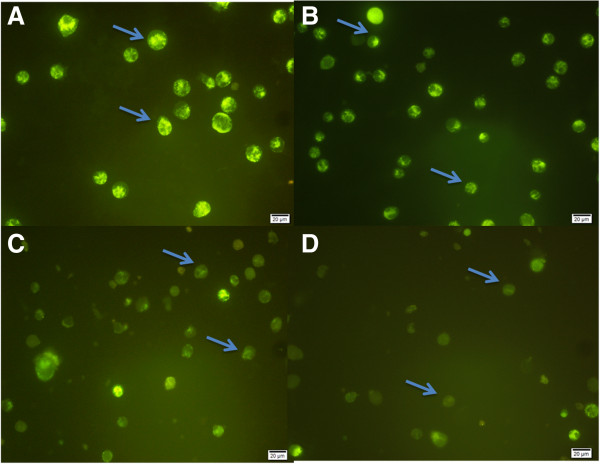
**Mitochondrial membrane potential analysis for CEMss cells treated with 8 μg/ml of Boesenbergin A for A).** Untreated cells showed bright green fluorescence color (arrow). **B**) CEMss cells after 24 h treatment showed decrease in fluorescence intensity (arrow). **C**) CEMss cells after 48 h treatment showed further decrease in fluorescence intensity (arrow). **D**) CEMss cells after 72 h treatment showed faded fluorescence intensity.

The mitochondria are an integral part of the apoptotic machinery and an event such as the loss of mitochondrial membrane potential (MMP) is classical evidence for apoptosis. To study changes in the mitochondria, the MMP was checked using fluorescence microscopy images on treated and untreated cells stained with Rh123 (Figure 8). The results clearly showed that the fluorescence intensity of Rh123 reduced as the treatment time increased. The bright green fluorescence of control cells (Figure 8A) was reduced significantly on boesenbergin A treatment (Figure 8D).

### Protein array analysis

**Figure 9:**
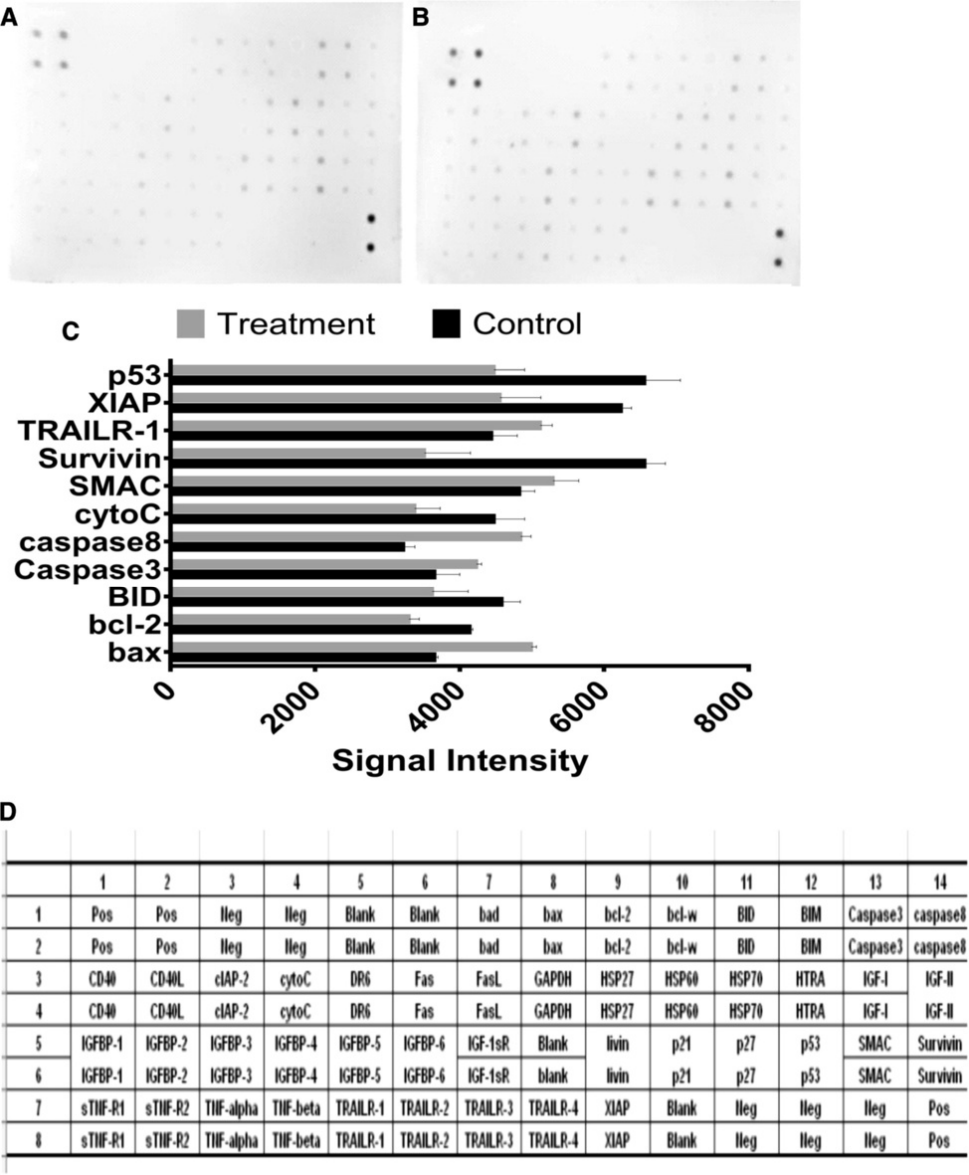
**Protein array analysis of CEMss untreated and treated cells for 72 h with 8 μg/ml of Boesenbergin A for A) Before treatment, B) After treatment and C) Histrogram. D**) The exact protein name of each dot in the array.

The pro-apoptotic protein, Bax was observed to be up-regulated, in CEMss cells which had been treated for 72 h with boesenbergin A (8 μg/ml), whereas both the anti-apoptotic protein, Bcl-2, and BID protein were found to be down-regulated. Both caspase-3 and =-8 increased substantially, further confirming the earlier assay results. Cytochrome c, survivin, XIAP and P53 protein decreased upon treatment, while TRAIL-R1 and SMAC proteins increased (Figure 9).

### Western blot analysis

**Figure 10:**
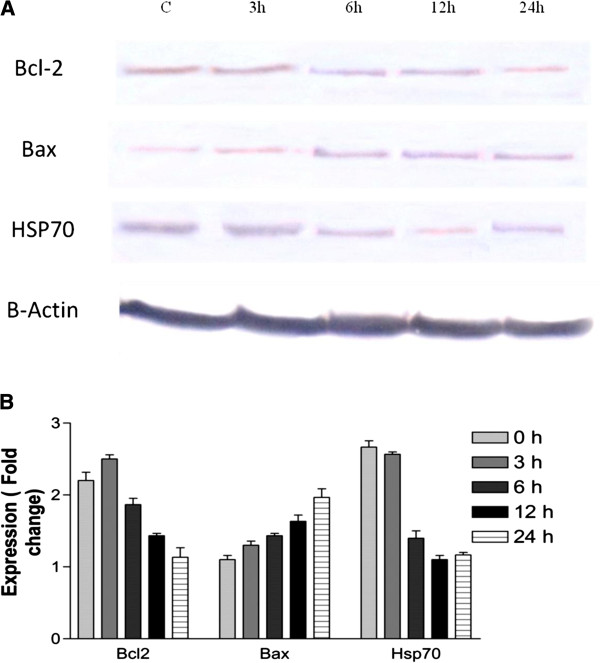
Effect of Boesenbergin A on the levels of apoptosis regulatory proteins at 3, 6, 9, and 12h with β- actin as a loading control.

To confirm the changes in proteins observed in protein array analysis and the presence of mitochondria in the apoptosis induced by boesenbergin A, we then evaluated the protein level using western blot analysis. Exposure of CEMss cells to boesenbergin A increased the expression of Bax and decreased the expression of Bcl2. Furthermore, the expression of HSP70 was down-regulated in a concentration dependent manner (Figure 10).

## Discussion

The rhizome of *Boesenbergia rotunda* is known to be able to treat a lot of ailments including colic, oral diseases, urinary disorders, dysentery and inflammation [5], however few studies have been carried out on the pure active compounds derived from this plant. Recently, it was found that boesenbergin A possesses cytotoxic activities against cancer cell lines including HepG2, HT-29, A549 and PC3 [15]. In this study, the MTT assay revealed that boesenbergin A had different degrees of cytotoxicity against MCF-7, Hela and CEMss cells (Table 1). with the IC_50_ for CEMss cells being found to be lower than for the other cells screened. Thus, in the present study, we focused on investigating the cytotoxic activity of boesenbergin A and its underlying mechanism of action against CEMss cells. One of the other benefits found from boesenbergin A is that it has no toxic effects against primary human blood lymphocytes.

Microscopic observations showed that, following treatment with boesembergin A, the numbers of viable CEMss cells were reduced with increasing treatment time. In addition, indications of apoptosis in treated CEMss cells such as cytoplasmic shrinkage and membrane blebbing were observed [19]*.* It was found that the number of cells undergoing apoptosis was more greater at earlier stages of treatment such as after 24 h and 48 h periods. However, when treatment time increased to 72 h, the presence of necrosis amongst treated CEMss cells was evident. This is possible since treated CEMss cells undergoing apoptosis may have progressed into necrosis due to the prolonged incubation with boesenbergin A. Treated CEMss cells showed morphological changes that included chromatin condensation, DNA fragmentation and membrane blebbing when observed under confocal microscopy using AO/PI staining. Following cell cycle analysis, we were able to further confirm the involvement of apoptosis in CEMss cells upon treatment with boesenbergin A in a time-dependent pattern, as well as observing that the compound induced cell cycle arrest at G_2_/M phase. Results from the Annexin V assay also point to the involvement of apoptosis in boesenbergin A treated CEMss cells. Similar findings have been reported that panduratin A from *Boesenbergia rotunda* also has the capability to arrest cells at the G_2_/M phase and induce apoptosis in PC3 and DU145 human prostate cancer cells [12] and A549 human non-small cell lung cancer cells [13].

In order to elucidate the mechanism of apoptosis, DNA laddering was performed. DNA fragmentation of CEMss cells was clearly detected after treatment with 16 μg/ml of boesenbergin A for 6 and 12 h. The ability to cause DNA fragmentation is one of the hallmarks of apoptotic cell death [20-22], including nuclear condensation and fragmentation, cleavage of chromosomal DNA into internucleosomal fragments and packaging of the dead cells into apoptotic bodies without plasma membrane breakdown. These features of apoptosis differ significantly from those of necrosis, which is morphologically characterized by vacuolation of the cytoplasm, breakdown of the plasma membrane and induction of inflammation around the dying cell, attributable to the release of cellular contents and pro-inflammatory molecules [20].

The caspase cascade signaling system is an important component in the process of apoptosis as it is controlled by various molecules that either enhance apoptosis or inhibit apoptosis. In this study, the levels of caspases =-3/7, -8, and =-9 were found to increase when CEMss cells were incubated with boesenbergin A. The activation of caspase-9 provides evidence that the compound is capable of triggering apoptosis via the mitochondrial pathway, whereas the increase in the caspase-3 level suggests that it can trigger DNA fragmentation [23]. The increase in the caspase-8 level is also an indication of apoptosis taking place since this caspase is involved in mediating Fas-induced apoptosis [24-27]. The increase in levels of caspase =-3/7, -8, and =-9 induced in the CEMss cells after treatment with boesenbergin A allows fragmentation of DNA to proceed towards cell death during apoptosis induction. The activation of caspase-9 has previously been found to be the step prior to the activation of caspase-3 in the activation cascade of the mitochondrial intrinsic pathway leading towards apoptosis [28]. This activation of caspase-9 is well controlled by the apoptosome, which converts procaspase-9 to caspase-9. The formation of the apoptosome is fully dependent upon the release of cytochrome c from the mitochondria to the cytosol and adhering to Apaf-1 [29]. Since the role of mitochondria in apoptosis is inevitable, we observed a reduction in MMP as the treatment time with boesenbergin A increased. This increase in mitochondria membrane permeability may be due to the up and down regulation of apoptosis proteins involved in the cell death mechanism.

There are a large number of proteins involved in the process of apoptosis [30,31]. In order to identify the contribution of central apoptosis proteins, we performed a protein array analysis. Several proteins in both the extrinsic and intrinsic pathways were investigated in the current study, including those known to induce apoptosis, such as Bax, caspase-3, caspase-8, SMAC and TRAILR-1 and those known to be anti-apoptotic, such as Bcl-2, X-linked IAP (XIAP) and surviving, where survivin and XIAP are members of the inhibitors of apoptosis (IAP) family of proteins previously [32] and SMAC is a pro-apoptosis protein that interacts with IAP to relieve their inhibitory effects [33,34].

The obtained protein array results showed a typical profile of protein levels associated with mitochondrial apoptosis in boesenbergin A treated CEMss cells. Hence we then selected the most important proteins involved in this pathway (Bax and Bcl-2) and conducted an immunoblot analysis. The expression of Bax showed an increase, whereas the expression of Bcl-2 was decreased, further confirming the protein array results. The decreased expression of Bcl-2 was expected since Bcl-2 prevents induction of apoptosis by blocking the release of cytochrome c from mitochondria [35]. Moreover, proteins of the Bcl-2 family are known to regulate the promotion and inhibition of apoptosis [36]. These Bcl-2 family proteins are highly expressed in CEMss cancer cells and therefore inhibiting its expression in the cancer cell will trigger cell death [37,38]. Along with Bcl-2 family members, heat shock proteins also have considered as apoptosis inhibitors, as they play a significant role in the survival of cells either my blocking the release of cytochrome c from mitochondria or by blocking the formation of apoptosome [39]. The immunoblot analysis demonstrated showed that Hsp70 was significantly reduced upon treatment with boesenbergin A. This correlates well to a previous study that showed that over-expression of Hsp70 was able to suppress apoptosis [40].

On the basis of the observations mentioned in this report, it can be concluded that the treatment of CEMss with boesenbergin A induced apoptosis with cell death-transducing signals that regulate the MMP by down-regulation of Bcl2 and up-regulation of Bax. Cell death was significantly controlled by both initiator and executioner caspases and resulting in the cleavage of specific substrates leading to the process of apoptotic changes. This form of apoptosis was found to be closely associated with the down regulation of Hsp70 and G2/M phase cell cycle arrest. The positive outcomes of our research provide a strong basis for developing boesenbergin A as a novel chemotherapeutic agent for leukemia intervention, which warrants further investigations including in animal models.

## Conclusion

In this current study, the results that we gather demonstrated that boesenbergin A induced apoptosis of CEMss cells through Bcl2/Bax signaling pathways with the involvement of caspases and G2/M phase cell cycle arrest. The current findings also warrant further research on boesenbergin A as a novel chemotherapeutic agent for leukemia intervention including studies in animal models.

## Competing interests

The authors declare that they have no competing interests.

## Authors’ contributions

KBN: Project design, experimental works, data analysis and manuscript preparation. AB and MAS: Boesenbergin A isolation and purification. SIA, SM and MJCB: Project design, data analysis and project coordination. BK, NMN and TA: Project design, statistical analysis, project coordination and manuscript preparation. AHAH and HSR: manuscript preparation, correction and isolation. All authors have read and approved the final version of the manuscript.

## Pre-publication history

The pre-publication history for this paper can be accessed here:

http://www.biomedcentral.com/1472-6882/13/41/prepub
